# Cardiovascular Benefits of GLP-1-BasedTherapies in Patients with Diabetes Mellitus Type 2: Effects on Endothelial and Vascular Dysfunction beyond Glycemic Control

**DOI:** 10.1155/2012/635472

**Published:** 2012-04-17

**Authors:** Thomas Forst, Matthias M. Weber, Andreas Pfützner

**Affiliations:** ^1^Institute for Clinical Research and Development, 55116 Mainz, Germany; ^2^First Medical Department, Johannes Gutenberg University, Mainz, Germany

## Abstract

Type 2 diabetes mellitus (T2DM) is a progressive multisystemic disease accompanied by vascular dysfunction and a tremendous increase in cardiovascular mortality. Numerous adipose-tissue-derived factors and beta cell dysfunction contribute to the increased cardiovascular risk in patients with T2DM. Nowadays, numerous pharmacological interventions are available to lower blood glucose levels in patients with type 2 diabetes. Beside more or less comparable glucose lowering efficacy, some of them have shown limited or probably even unfavorable effects on the cardiovascular system and overall mortality. Recently, incretin-based therapies (GLP-1 receptor agonists and DPP-IV inhibitors) have been introduced in the treatment of T2DM. Beside the effects of GLP-1 on insulin secretion, glucagon secretion, and gastrointestinal motility, recent studies suggested a couple of direct cardiovascular effects of GLP-1-based therapies. The goal of this paper is to provide an overview about the current knowledge of direct GLP-1 effects on endothelial and vascular function and potential consequences on the cardiovascular outcome in patients with T2DM treated with GLP-1 receptor agonists or DPP-IV inhibitors.

## 1. Introduction

The global prevalence of T2DM has been estimated at 171 million people and is projected to more than double by 2030 [1]. The epidemiological establishment of T2DM as a coronary artery disease equivalent has been confirmed in several investigations [2–4]. Type 2 diabetes mellitus (T2DM) is a progressive multisystemic disease accompanied by endothelial dysfunction [5–7] and an increased cardiovascular mortality [3, 8]. Also several mechanistic pathways linking glucose metabolism with endothelial dysfunction are postulated; recent studies aimed to investigate the beneficial role of strict glycemic control using conventional treatment algorhythms failed to provide beneficial effects on cardiovascular mortality [9–11]. The accumulation and interaction of several metabolic and cardiovascular risk factors merge in the pathophysiology of diabetic vascular disease and the development of micro- and macrovascular complications. Diabetic vascular disease has been associated with an upregulation of reactive oxygen species and chronic inflammatory and hypercoagulable states, and as such with the pathogenesis of atherosclerosis and cardiovascular disease.

## 2. Adipose Tissue, Insulin Resistance, and Beta Cell Function in Vascular Pathology

**Figure 1:**
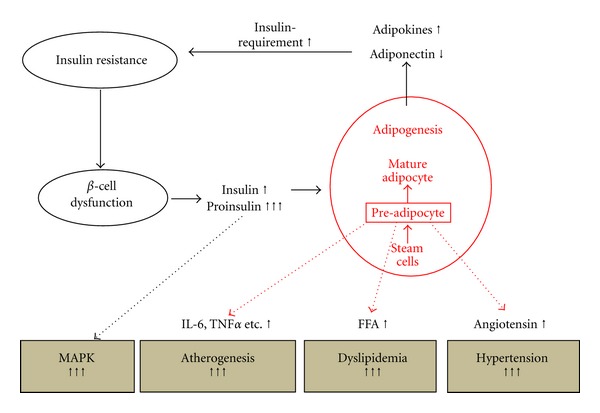
Schematic illustration of the central role of visceral adipose tissue in the generation of the atherogenic environment in obese patients. Visceral adipose tissue induces insulin resistance, thereby increasing insulin and proinsulin release from the beta cell. Unphysiological levels of insulin and proinsulin promote atherogenesis through the activation of endothelial MAPK. Preadipocytes secrete numerous adipocytokines involved in the pathogenesis of hypertension, dyslipidemia, and inflammation. (MAPK: mitogen-activated protein kinase, IL-6: interleukin-6, TNF**α**: tumor necrosis factor *α*, FFA: free fatty acids).

Adipose tissue is assumed to play an integral role in the pathogenesis of vascular dysfunction and the development of T2DM [12]. As maturing pre-adipocytes differentiate to become adult adipocytes, they acquire the ability to synthesize hundreds of proteins. Adipose tissue releases a large number of cytokines and bioactive mediators with endocrine, autocrine, juxtacrine, and paracrine activity. These proinflammatory adipocytokines include TNF-*α*, IL-6, leptin, plasminogen activator inhibitor 1 (PAI-1), angiotensinogen, resistin, and c-reactive protein, all of them well known to interfere with insulin sensitivity, blood pressure regulation, lipid metabolism, and inflammation (Figure 1). On the other hand, the release of adiponectin, an anti-inflammatory and vasoprotective adipokine, is reduced in insulin-resistant obese patients and in patients with T2DM [13]. In addition, it is likely that many more undiscovered fat cell-derived mediators are causally involved in cardiovascular health, insulin resistance, and the development of T2DM.

Epidemiological evidence suggests that cardiovascular risk begins to develop many years before the diagnosis of T2DM [14]. Increasing insulin resistance, associated with increasing visceral body fat, is associated with an increased risk for cardiovascular disease and is commonly comorbid with hypertension, dylipidemia, obesity, and a prothrombotic state [15–17]. As long as the pancreatic beta cells compensate for increasing insulin resistance with an augmented release of insulin, blood glucose will stay controlled, and the patient will not present, as per definition, with frank diabetes mellitus. There are strong prospective data that, even before deranging glucose levels, obesity and insulin resistance are associated with atherosclerosis and coronary heart disease [18, 19]. However, it is the progressively deteriorating function of the beta cell, which ultimately leads to hyperglycemia and is critical for the manifestation of T2DM. There is increasing evidence that islet cell polymorphisms my account for the individual risk for beta cell breakdown and the manifestation of T2DM as defined by an increase in fasting and/or postprandial blood glucose levels [20, 21]. The insulin/proinsulin ratio is used as a marker for the capability of the *β* cell to convert intact proinsulin into insulin and C-peptide [21]. Preclinical and clinical studies of type 2 diabetes have identified proinsulin both as an indicator of decreasing beta cell function and a predictor of increased beta cell loss due to apoptosis and/or diminished neogenesis [21, 22]. Furthermore, a direct role of this prohormone in the development of cardiovascular disease has been suggested by numerous experimental and epidemiological studies. Increased intact proinsulin levels were found to be closely associated with the development of coronary heart disease involving subjects with and without diabetes [23–27]. In fact, the atherogenic potential of proinsulin was highlighted some years ago in a clinical trial, investigating the therapeutic potential of human proinsulin given as subcutaneous injections. In that study, an eightfold increase in cardiovascular events was observed during treatment with human proinsulin versus human regular insulin [28]. More recently, an association between increased plasma concentrations of proinsulin and the severity of angiographically characterized coronary heart disease has been reported [29]. Even the exact mechanism how proinsulin is involved in the pathogenesis of atherosclerosis is not completely recognized, it was already shown that PAI-1 activity increases after proinsulin administration in vitro [30]. Increased expression of PAI-1 and vascular adhesion molecules have been associated with hyperglycemia-related endothelial cell dysfunction and a predisposition to accelerated atherogenesis [31]. There is increasing evidence that the atherogenic effects of proinsulin might, at least in part, be mediated by increasing PAI-1 levels with subsequent inhibition of fibrinolysis and an augmented thrombogenic potency [32–34]. In accordance with this finding, the reduction of intact proinsulin levels during treatment with a PPAR*γ* agonist in T2DM was shown to be associated with a decrease in intima media thickness of the arteria carotis communis [35].

## 3. Endothelial Dysfunction in Diabetes Mellitus

Vascular wall dysfunction is a critical mediator of atherogenesis in patients with T2DM. The response to injury hypotheses of atherosclerosis supposed that the initial damage affects the arterial endothelium leading to endothelial dysfunction [36]. Endothelial dysfunction in patients with obesity and T2DM is characterized by an imbalance between endothelium-dependent vasodilatation and vasoconstriction as well as antithrombotic and prothrombotic factors. Nitric oxide (NO) maintains the vasodilatation and vasoprotective property of the endothelium and opposes the effects of vasoconstrictors such as endothelin 1 or angiotensin II [6, 37]. It inhibits leucocyte and platelet activation and helps to maintain the endothelium as smooth nonthrombotic barrier. Thus endothelial dysfunction is a prominent feature at various stages of atherogenesis, vessel occlusion, and tissue infarction [12]. In a study by Goldfine et al., endothelial function and NO bioavailability was evaluated in 38 individuals without a history of T2DM [38]. In this study, 19 patients were offspring of type 2 diabetes parents, while the other 19 had no first degree relatives with either T2DM or cardiovascular disease. Patients with a family history of diabetes were found to have significantly reduced endothelial function and nitric oxide (NO) bioavailability.

**Figure 2:**
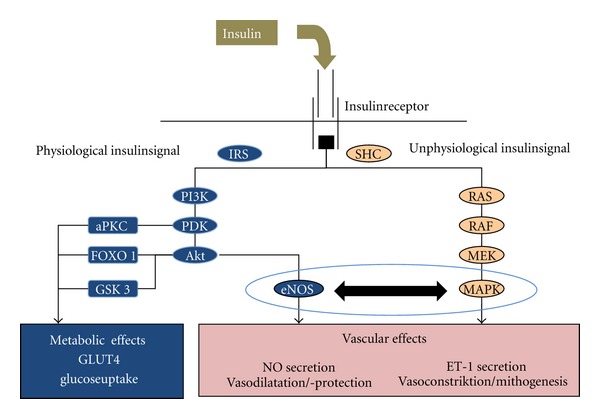
Schematic illustration of intracellular insulin signalling (aPKC: atypical Protein-Kinase C, eNOS: endothelial Nitric Oxide Synthase, ET1: Endothelin 1, FOX01: Forkhead Box01, IRS: Insulin Receptor Substrate, MAPK: Mitogen Activated Proteinkinase, MEK: Mitogen Activated Proteinkinase Kinase, NO: Nitric Oxide, PDK: Phosphoinositide-Dependent Kinase, PI3K: Phosphatidylinostol 3 Kinase, RAS, RAF: Proto-Oncogenes, SHC: Adapter Protein; adapted to [6]).

Beside the well-known metabolic effects of insulin, the hormone exerts important vascular effects through the activation of opposing intracellular signaling pathways. Under normal conditions, insulin provides vasodilatation and vasoprotective effects through the activation of the Phosphoinositol-3-Kinase pathway (PI-3 K) and promoting the release of NO. In contrast, in case of insulin resistance and an unphysiological release of insulin and proinsulin from the beta cells, the signal shifts from the vasoprotective PI-3 K pathway to the mitogen activated proteinkinase (MAPK) pathway (Figure 2). In this case, the vasodilating and beneficial effects of insulin turn over into the vasoconstrictive and mitogenic properties of insulin [6, 39].

## 4. Effects of GLP-1 on Body Weight and Beta Cell Function

Nowadays, numerous pharmacological interventions are available to lower blood glucose levels in patients with type 2 diabetes. Beside more or less comparable glucose lowering efficacy, some of them have shown limited or probably even unfavorable effects on the cardiovascular system and overall mortality [40–44]. Therefore, treatments in T2DM should be reevaluated not solely by judging their glucose lowering potency, but, moreover, on their overall effects on the cardiovascular risk profile and overall mortality in patients with T2DM.

Recently, incretin-based therapies have been introduced in the treatment of T2DM. Incretins, gut-derived hormones, predominantly glucagon peptide 1 (GLP-1), and gastric inhibitory polypeptide (GIP), are released in response to an ingested meal. They reduce blood glucose concentrations by enhancing the insulin release from pancreatic beta cells [45] and inhibiting postprandial glucagon secretion [46, 47] and gastric emptying after a meal [48]. However, only GLP-1 seemed to be suitable for the treatment of type 2 diabetic patients since the action of GIP was found to be grossly impaired in patients with T2DM [49]. Following its release from the L-cells into the blood stream, GLP-1 [7–36] is quickly cleaved by the enzyme dipeptidyl-peptidase-IV (DPP-IV) into the split product GLP-1 [9–39], which results in a half-life time of GLP-1 [7–39] of only 2 minutes. Therefore, strategies have been developed to increase the therapeutical window of GLP-1-based treatments by inhibiting the degrading enzyme DPP-IV or by providing exogenous agonists of GLP-1, which are resistant to the degradation by DPP-IV. At present, four DPP-IV inhibitors (Sitagliptin, Vildagliptin, Saxagliptin, and Linagliptin) and two GLP-1 receptor agonists (Exenatide and Liraglutide) are approved for the treatment of T2DM. In several studies, treatment with DPP-IV inhibitors and GLP-1 receptor agonists was shown to improve blood glucose control in type 2 diabetic patients without increasing the risk of hypoglycemia. While treatment with DPP-IV inhibitors was shown to be more or less weight neutral, treatment with the GLP-1 receptor agonists reduced body weight in the majority of patients with T2DM [50]. Beside these overall weight reducing effects of GLP-1 receptor agonists a pronounced reduction in visceral body fat was shown during treatment with Liraglutide [51]. In another study, treatment with the GLP-1 receptor agonist Exenatide resulted in a reduction in body weight, waist circumference, total body, and truncal fat mass by 6%, 5%, 11%, and 13%, respectively [52]. In addition, exenatide increased total adiponectin by 12% and reduced high-sensitive CRP by 61%. Treatment with GLP-1 receptor agonists has been consistently demonstrated to reduce blood pressure, as observed in clinical trials with exenatide or liraglutide [53–57]. Of interest is, that the reduction of blood pressure in most studies occurred even before weight reduction could be achieved, indicating direct effects of GLP-1 on blood pressure. The mechanisms beyond the blood pressure lowering effects of GLP-1 are still unclear. Beside changes in the release of adipokines from the adipose tissue, direct vasodilatory effects [58] and renal natriuretic effects [59] of GLP-1 might cause the beneficial effects of GLP-1 on blood pressure.

**Figure 3:**
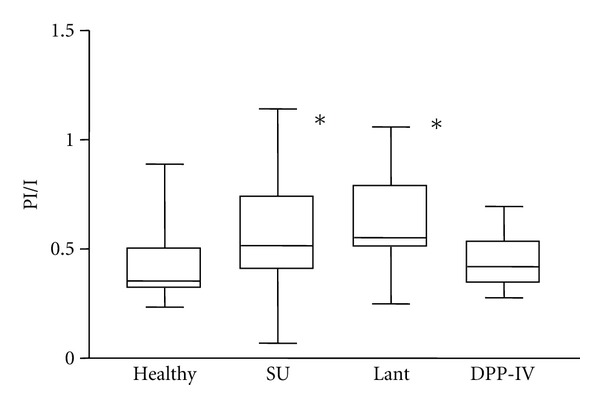
Postprandial proinsulin/insulin ratio in patients treated with sulfonylurea, insulin Glargine, or DPP-IV inhibitors compared to nondiabetic healthy controls (*: *P* < 0.05 versus nondiabetic controls adapted to [67]).

Recently, it has been reported that the vulnerability of the beta cell to release the prohormone intact proinsulin, instead of insulin and C-peptide, is linked to beta cell polymorphisms observed in three different risk alleles [22]. The mechanisms by which these risk alleles (HHEX, CDKN2A/B, and IGF2BP2) influence proinsulin processing are still a matter of debate. Loos et al. demonstrated that the genes of pro-protein convertases 1 and 2, which are key proteins in the conversion from proinsulin to insulin, exhibit binding sites for the *T*-cell transcription factor [60]. Therefore, an interaction with these pro-protein convertases may be a mechanism leading to increased proinsulin levels in carriers of the risk alleles [22]. As GLP-1 infusion is able to normalize reduced proinsulin conversion, and GLP-1 signaling is impaired in carriers of the risk alleles in TCF7L2, it is conceivable that an attenuated GLP-1 signaling might lead to an impaired proinsulin processing [61]. Several clinical studies have shown an improvement in the Proinsulin/Insulin Ratio during treatment with GLP-1 receptor agonists [62, 63] and DPP-IV inhibitors [64–66]. In a cross-sectional study, postprandial intact proinsulin levels were significantly higher in sulfonylurea-treated patients compared to insulin and DPP-IV-inhibitor-treated patients and a non-diabetic control group [67]. As shown in Figure 3, the proinsulin/insulin ratio was comparable in between the DPP-IV-inhibitor-treated group and the nondiabetic control group, while it was evaluated in the sulfonylurea- and the insulin-treated group. In a recent study, the introduction of liraglutide treatment caused a pronounced decrease in intact proinsulin levels, which was found in parallel with a reduction in E-Selectin, asymmetric-dimethyl-arginine (ADMA), and PAI-1 levels, and an improvement in endothelial function [68]. Therefore, it seems conceivable that GLP-1-based treatments do not only improve beta cell function by an induction of the proinsulin convertases but also reduce vascular risk by the reduction in circulating absolute proinsulin levels.

## 5. Effects of GLP-1 on Endothelial and Cardiovascular Function

**Figure 4:**
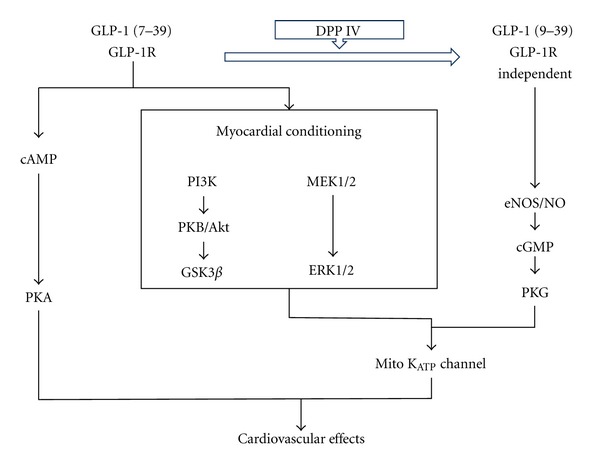
Schematic illustration of GLP-1-receptor-dependent and GLP-1-receptor-independent pathways in the signalling of GLP-1 associated cardiovascular effects (PI3K: phosphatidylinositol 3 kinase; cAMP: cyclic adenosine monophosphate, PKA: protein kinase A, PKB: protein kinase B, GSK3ß: glycogen synthase kinase 3 beta, MEK1/2: components of the MAP kinase cascade, ERK: extracellular-signal-regulated kinase, NO: nitric oxide, eNOS: endothelial nitric oxide synthase, cGMP: cyclic guanosine monophosphate, PKG: protein kinase G; adapted to [69]).

GLP-1 acts through a distinct heptahelical G-protein-coupled receptor, which has been located not only in beta cells and the gastrointestinal tract, but also in the nervous system, heart, vascular smooth muscle cells, endothelial cells, and macrophages [45, 58, 70, 71]. GLP-1 appears to modulate a wide range of physiological effects, not only regulating blood glucose and metabolic control, but also directly affecting several cardiovascular pathways involved in atherogenesis. Binding of GLP-1 to the GLP-1 receptor in the myocardium leads to an increase in the production of cyclic adenosine monophosphate (cAMP) and an activation of protein kinase A (PKA), which results in an increase in glucose uptake and the inotropic effects in myocardial tissue. GLP-1 knockout mice exhibit reduced resting heart rate, elevated left ventricular and diastolic pressure, and increased left ventricular thickness compared with normal controls [58]. In agreement with this findings, treatment with GLP-1 or the GLP-1 receptor agonists was found to improve left ventricular function [72, 73] and to reduce circulating levels of brain natriuretic peptide (BNP) [74, 75]. GLP-1 increased functional cardiomyocyte viability in an isolated mouse heart reperfusion model but, unexpectedly, many of these actions were preserved in the GLP-1 knockout mouse model, suggesting cardiovascular effects that are independent of the GLP-1-receptor-mediated activation of cAMP/PKA [50]. Ban et al. studied the myocardial effects of GLP-1 (7-39) and GLP-1(9-39) under postischemic conditions in both wild-type and GLP1r (−/−) (GLP-1 receptor knockout mice) [58]. In wild-type mice, some of the effects of GLP-1 (7-39) could be reproduced by the truncated peptide GLP-1 (9-39). Those effects could also be induced by GLP1 (7-39) and GLP-1 (9-39) in the GLP1r (−/−) knockout mouse model. In addition, the beneficial effects of GLP-1 in the knockout mouse model could be abolished by the addition of the DPP-IV inhibitor Sitagliptin and by the administration of the NO-synthase blocker L-NNA (N^G^-nitro-arginine). This leads to the hypothesis of a two pathway mechanism for the protective cardiovascular action of GLP-1. One that depends on the GLP-1 receptor mediated action with inotropic, glucose uptake stimulating, ischemic preconditioning effects, and one, receptor independent vasodilatatory pathway, which is mediated by NO (Figure 4).

Growing lines of evidence demonstrate beneficial effects of GLP-1 on endothelium and vascular smooth muscle cells [58, 76–78]. In human vascular endothelial cells, liraglutide caused eNOS phosphorylation and potentiated eNOS activity with an increased nitric oxide production [79]. In endothelial cells, isolated from human coronary arteries, GLP-1 rapidly activates endothelial nitric oxide synthase (eNOS), promotes cell proliferation, and inhibits glucolipoapoptosis [80]. In healthy, nondiabetic, subjects, GLP-1 infusion enhanced the acetylcholine-mediated increase in forearm blood flow, while no such effect could be observed on endothelial-independent vasodilatation after sodium nitroprusside [78]. Interestingly, in that study, the beneficial effects of GLP-1 on endothelial function were damped by the addition of glyburide, but not by glimepiride. In type 2 diabetic patient with coronary artery disease, infusion of GLP-1 increased flow-mediated vasodilatation in the brachial artery, affirming the NO-dependent mechanism of GLP-1 in the vascular system [81]. In this study, no such effect could be observed in nondiabetic healthy controls.

## 6. Other Potential Cardiovascular Benefits

Treatment with the GLP-1 receptor agonists, liraglutide and exenatide, was shown to reduce PAI-1 levels in patients with T2DM [68, 74, 75, 82]. In cultured human vascular endothelial cells, Liraglutide inhibited the expression of tumor necrosis factor-*α* (TNF *α*) and the hyperglycemia-mediated induction of VCAM-1 and PAI-1 [74, 80]. This may be of relevance as elevated PAI-1 levels have been implicated in endothelial cell dysfunction [83]. In a previous study, 14 weeks of treatment with liraglutide significantly decreased PAI-1 levels by 25% [74]. Complementing the beneficial effect on PAI-1 levels, liraglutide attenuates the induction of PAI-1 and vascular adhesion molecules in vitro [31].

A recent metaanalysis of the 6 trials from the LEAD program revealed that treatment with the GLP-1 receptor agonist liraglutide decreased hsCRP levels by 23% from baseline till 6 months [84]. In a study comparing exenatide and glargine treatment, treatment with exenatide over a period of 12 months reduces hsCRP levels by 61% [52].

In addition to the effects on myocardium and the endothelial cells, GLP-1 may also have effects on atherogenesis through direct interactions with monocytes or macrophages. Treatment with Exenatide significantly inhibited monocyte adhesion in the aorta of C57BL/6 mice [85]. In apoE(−/−) mice, the same treatment reduced monocyte adhesion to the endothelium and suppressed atherogenesis.

As pointed out by Zilversmit many years ago, atherosclerosis could be considered to be a prandial phenomenon [86]. Therefore, GLP-1 might serve as a metabolic and vasoprotective factor evolving its main effects after the ingestion of a meal. In a study by Koska et al. postprandial endothelial function was investigated in IGT subjects and in patients with recently diagnosed T2DM by reactive hyperemia peripheral arterial tonometry [87]. In that study, a single dose of Exenatide resulted in a significant improvement in postprandial endothelial function compared with placebo administration. In another study, intravenous GLP (7-39) infusion resulted in a significant improvement of postprandial FMD during an OGT and during a hyperglycemic clamp procedure [88]. The improvement in FMD was paralleled by a reduced postprandial increase in nitrotyrosine and 8-iso-PGF2a levels. These results suggest that GLP-1 has the potential to reduce glucose load, oxidative stress and to improve endothelial function especially in the postprandial state.

How far all the previously observed pleiotropic effects of GLP-1 will translate in a reduction of micro- and/or macrovascular complications in patients with T2DM is still not established. In a retrospective analysis of the LifeLink database, patients treated with the GLP-1 receptor agonist Exenatide were less likely to have a CVD event (HR 0.81, *P* = 0.01) and lower rates of CVD-related hospitalization (HR 0.88, *P* = 0.02) [89]. A recent meta-analysis assessing the cardiovascular outcome of GLP-1-receptor agonists in clinical trials up to November 2010 revealed a significant lower rate of major cardiovascular events in GLP-1-receptor-agonist-treated patients compared to placebo [90]. Ongoing randomized prospective clinical studies will provide more evidence about potential clinical long-term effects of GLP-receptor agonist or DPP-IV inhibitor treatment in patients with T2DM at cardiovascular risk.
